# Ring-Opening Metathesis Polymerization and Related Olefin Metathesis Reactions in Benzotrifluoride as an Environmentally Advantageous Medium †

**DOI:** 10.3390/ijms24010671

**Published:** 2022-12-30

**Authors:** Ervin Kovács, Bence Balterer, Nguyen Anh Duc, Györgyi Szarka, Michael C. Owen, Attila Domján, Béla Iván

**Affiliations:** 1 Polymer Chemistry and Physics Research Group, Institute of Materials and Environmental Chemistry, Research Centre for Natural Sciences, Magyar Tudósok Krt. 2, H-1117 Budapest, Hungary; 2 NMR Research Laboratory, Centre for Structural Science, Research Centre for Natural Sciences, Magyar Tudósok Krt. 2, H-1117 Budapest, Hungary; 3 Hevesy György PhD School of Chemistry, Eötvös Loránd University, Pázmány Sétány 1, H-1117 Budapest, Hungary; 4 Institute of Chemistry, University of Miskolc, H-3515 Miskolc-Egyetemváros, Hungary

**Keywords:** olefin metathesis, ring-opening metathesis polymerization (ROMP), norbornene, ring-closing metathesis (RCM), isomerization, homogenous catalysis, benzotrifluoride, green solvent

## Abstract

A tremendous number of solvents, either as liquids or vapors, contaminate the environment on a daily basis worldwide. Olefin metathesis, which has been widely used as high-yielding protocols for ring-opening metathesis polymerization (ROMP), ring-closing metathesis (RCM), and isomerization reactions, is typically performed in toxic and volatile solvents such as dichloromethane. In this study, the results of our systematic experiments with the Grubbs G1, G2, and Hoveyda-Grubbs HG2 catalysts proved that benzotrifluoride (BTF) can replace dichloromethane (DCM) in these reactions, providing high yields and similar or even higher reaction rates in certain cases. The ROMP of norbornene resulted not only in high yields but also in polynorbornenes with a high molecular weight at low catalyst loadings. Ring-closing metathesis (RCM) experiments proved that, with the exception of the G1 catalyst, RCM occurs with similar high efficiencies in BTF as in DCM. It was found that isomerization of (Z)-but-2-ene-1,4-diyl diacetate with the G2 and HG2 catalysts proceeds at significantly higher initial rates in BTF than in DCM, leading to rapid isomerization with high yields in a short time. Overall, BTF is a suitable solvent for olefin metathesis, such as polymer syntheses by ROMP and the ring-closing and isomerization reactions.

## 1. Introduction

Olefin metathesis, developed more than three decades ago, is a widely used reaction for the formation of carbon-carbon double bonds [1,2,3]. Although this process has been utilized for the preparation of a large variety of polymers and low molecular weight substances since its inception (see, e.g., references [3,4,5,6,7,8,9,10,11,12,13,14,15,16,17,18,19,20,21] and references therein), metathesis reactions still belong to an extensively investigated research field. This is mainly due to the high efficiency of this reaction under relatively mild conditions. Therefore, olefin metathesis is a real research and development challenge in both academia and the chemical industry, especially for the preparation of polymers and large cyclic compounds by ring-opening metathesis polymerization (ROMP) and ring-closing metathesis (RCM), respectively. Ring-opening, in addition to cross metathesis resulting in unsaturated compounds, is also a highly relevant tool for the production of pharmaceuticals [18,19,20]. The first catalysts were very sensitive to air and moisture and required non-coordinating, special, and extra-pure solvents without nitrogen moiety and hydroxyl groups. Therefore, mostly dichloromethane, aromatic solvents, and tetrahydrofuran have been applied as reaction media for metathesis transformations [3,4,5,6,7,8,9,10,11,12,13,14,15,16,17,18,19,20,21], even in the case of ROMP of fluorous monomers [7].

Over the last twenty years, olefin metathesis research has been mostly focused on the design of more efficient, robust, less sensitive, and selective catalysts, as well as on the synthesis of a variety of special macromolecular materials and active pharmaceutical ingredients. Despite being a selective and intrinsically “green” catalytic process by using small amounts of catalysts, much less attention has been paid to finding environmentally more advantageous solvents as reaction media [22,23,24,25,26,27,28,29,30,31,32,33,34,35,36,37,38,39,40,41,42,43,44,45].

Some green solvents, such as glycerol [39], poly(ethylene glycol) [40,41], acetic acid [22], non-conventional ethyl lactate [42], dialkyl carbonates [43,44,45], D-lymonene [24], and supercritical carbon dioxide [25,26,27,28,29,30,31], were successfully applied as reaction media in certain olefin metathesis reactions. In some special cases, using mostly novel, non-commercially available catalysts, higher conversion can be achieved in solvents like ethyl acetate [37,38,43], 2-methyltetrahydrofuran [32,33,35,43], and 4-methyltetrahydropyran [36] than that in the commonly used dichloromethane, THF, or toluene.

**Scheme 1:**
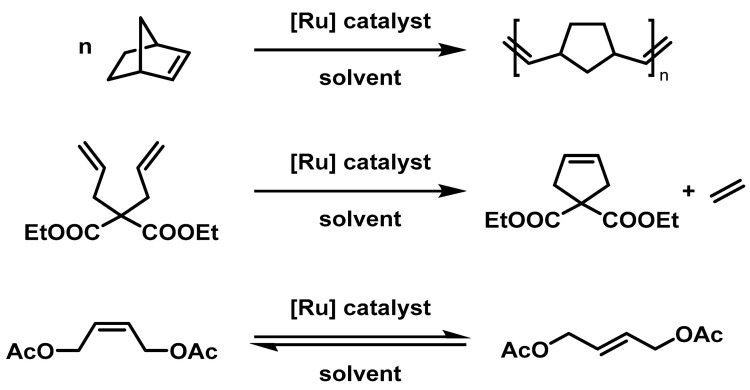
Ring-opening metathesis polymerization (ROMP) of norbornene (top), ring-closing metathesis (RCM) of diethyl 2,2-diallylmalonate (middle), and the isomerization reaction of (Z)-but-2-ene-1,4-diyl diacetate (bottom) by using ruthenium catalysts.

It has been found by us that one of the possible replacements of the environmentally harmful widely used solvents such as dichloromethane, tetrahydrofuran, and toluene, is benzotrifluoride (BTF), trifluoromethylbenzene, and α,α,α-trifluorotoluene) which has not been reported as a reaction medium for olefin metathesis yet. BTF is a solvent that is considered to be less harmful to the environment than many conventional reaction media [46,47,48]. It is a relatively inert compound and is suitable for a wide range of chemical transformations, including very sensitive thermal, ionic, and radical reactions that involve transition metal catalysts [46,47,48,49,50,51]. BTF has several advantageous properties compared to more toxic halogenated solvents such as dichloromethane, chloroform, dichloroethane, or to the aromatic solvents such as benzene, toluene, xylene, or even the peroxide-forming THF [46,47,48,49,50]. The polarity of BTF is similar to that of dichloromethane, but BTF has a significantly higher boiling point and a lower vapor pressure. Due to its commercial availability, low volatility, and low toxicity, BTF can be considered an environmentally advantageous replacement for the more widely used and more environmentally hazardous solvents mentioned above. It has already been applied in various organic synthetic reactions that were previously conducted in dichloromethane, toluene, or nitrobenzene [46,47,48,49,50,51,52,53]. Benzotrifluoride has also been used as a solvent for a variety of polymerization reactions, not only for the polymerization of various fluorous monomers [54], but also for the cationic polymerization of β-pinene [55], isoprene [56], styrene [57,58], isobutylene [57], and 2-ethyl-2-oxazoline [59]. The quasiliving atom transfer radical polymerization (ATRP) of styrene and n-butyl acrylate [57] in BTF has also been carried out successfully. However, to the best of our knowledge, there are no studies that have investigated the use of benzotrifluoride as a medium for olefin metathesis reactions. Herein, we report on the suitability of benzotrifluoride as a potential environmentally advantageous solvent for olefin metathesis. This is tested in three types of metathesis reactions: the ring-opening metathesis polymerization of norbornene, the ring-closing metathesis of diethyl diallyl malonate, and the isomerization of (Z)-but-2-ene-1,4-diyl diacetate (Scheme 1). The reaction kinetics and turnover number of these reactions in benzotrifluoride are compared to those carried out in dichloromethane, currently the most popular but toxic and environmentally hazardous solvent used for olefin metathesis.

## 2. Results and Discussion

**Scheme 2:**
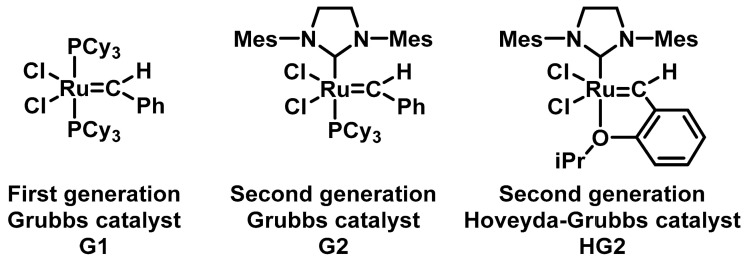
The olefin metathesis catalysts used in this study (Cy: cyclohexyl and Mes: mesityl, 2,4,6-trimethylphenyl group).

As shown in Scheme 1, three different kinds of olefin metathesis reactions—that is, the ring-opening metathesis polymerization (ROMP), the ring-closing metathesis (RCM), and the isomerization—were selected to be investigated by using benzotrifluoride (BTF) and dichloromethane (DCM) for comparison as reaction media for these reactions. The applied catalysts are displayed in Scheme 2. These include the 1st generation Grubbs (G1), 2nd generation Grubbs (G2), and the 2nd generation Hoveyda–Grubbs (HG2) catalysts.

### 2.1. Ring-Opening Metathesis Polymerization of Norbornene in Benzotrifluoride

**Figure 1:**
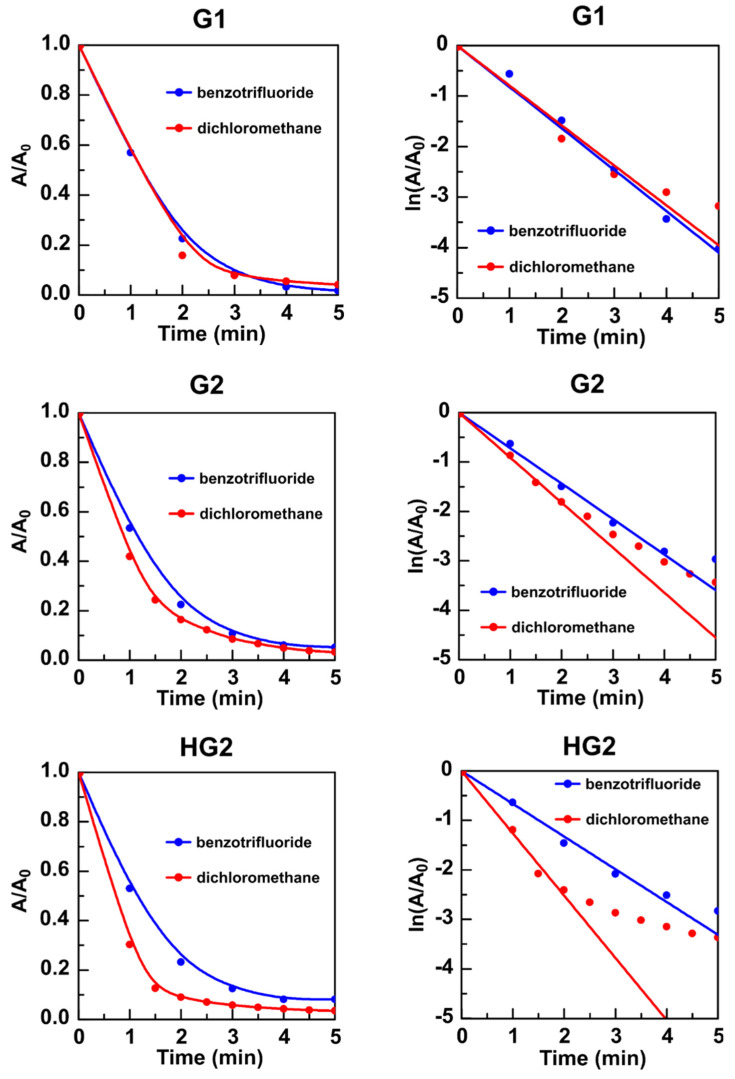
The monomer consumption (A/A_0_) as a function of time and its first order plots for the ring-opening metathesis polymerization of norbornene (c_0_ = 106 mM) in benzotrifluoride and in dichloromethane in the presence of 1st generation Grubbs (G1, 0.05 mol%), 2nd generation Grubbs (G2, 0.01 mol%), and 2nd generation Hoveyda-Grubbs (HG2 0.003 mol%) catalysts at 25 °C (A: ^1^H NMR signal integral of the vinylic hydrogen signal of norbornene; A_0_: initial ^1^H NMR signal integral of the vinylic hydrogen signal of norbornene; see Figures S2 and S3).

For investigating the effect of BTF in comparison with DCM in ROMP, the highly strained bridged cyclic hydrocarbon norbornene was chosen as a monomer (Scheme 1, top). These polymerization reactions, catalyzed by 0.05 mol% G1, 0.01 mol% G2, and 0.003 mol% HG2 ruthenium-based catalysts, were carried out at 25 °C. The monomer consumption versus time curves were recorded for each catalyst in both solvents (Figure 1). ^1^H NMR spectroscopy was used to monitor the monomer consumption by following the decrease of the vinylic hydrogen signal of norbornene in each reaction over time [60]. As shown in Figure 1 and Table 1, the monomer consumption versus time curves and the rate constants (k_1_) and half-lives (t_1/2_) for the ROMP reaction in benzotrifluoride are nearly identical to those accomplished in dichloromethane. For easy comparison, the first-order plots of the monomer consumption were also depicted, and the data from the first three minutes were used to determine the k_1_ rate constant. The monomer conversions were greater than 99% within ten minutes in all the cases (Figure 1). The rates of polymerization are comparable in the cases of the G1 and G2 catalysts in both solvents. In the case of the HG2 catalyst, the rate of the polymerization and thus the rate constant of the ring-opening metathesis polymerization of norbornene in BTF is smaller than that measured in DCM, as shown in Figure 1 and Table 1.

High molecular weight polynorbornenes are formed by ROMP in both benzotrifluoride and dichloromethane, as shown in Table 1 and Figure S1. The average molecular weights (M_n_) of the resulting polynorbornenes depend on both the solvents and the catalysts. On the one hand, polymers with higher Mn content are obtained in DCM than in BTF. On the other hand, polynorbornenes with the highest molecular weights are formed with the highly active HG2 catalyst in both solvents.

The performance of the catalysts was also compared by the turnover number. As shown in Table 2, the turnover numbers of the ring-opening metathesis polymerization of norbornene are greater than 480,000 when the G2 or HG2 catalysts are used in both BTF and DCM, whereas they are 385,100 when the less reactive G1 catalyst [61] is used in BTF. This turnover number with the G1 catalyst in BTF can still be considered high; however, in the rare cases when an even higher turnover number is needed, the more effective G2 or HG2 catalysts can be used instead. In conclusion, there is no significant difference in turnover numbers, which proves that benzotrifluoride is an alternative solvent for the hazardous dichloromethane for the ring-opening metathesis polymerizations with all the investigated catalysts applied at very low loadings.

The excellent performance of BTF compared to the environmentally hazardous DCM was achieved under the standardized olefin metathesis conditions, which include using an inert nitrogen atmosphere and freshly distilled solvents for each reaction. In order to reveal the effect of the purification (distillation) of BTF and the atmosphere, i.e., nitrogen or air, polymerizations were carried out in unpurified crude BTF as well as in air. As shown in Table S1, there is no significant difference in the effectiveness of the G2 catalyst when the reactions are performed in either distilled or crude BTF. However, the G1 and HG2 catalysts are somewhat more sensitive to the impurities, such as moisture, which may be present in the crude BTF.

The impact of oxygen on the emerging Ru-carbene catalysts for olefin metathesis was reviewed in recent years [62,63,64]. Not surprisingly, if the experiments involving BTF are carried out in air, the performance of catalysts is poorer than under nitrogen in all cases (Table S1). Therefore, a dry nitrogen atmosphere is preferred to carry ROMP with high catalyst efficiency in BTF.

### 2.2. Ring-Closing Metathesis

**Figure 2:**
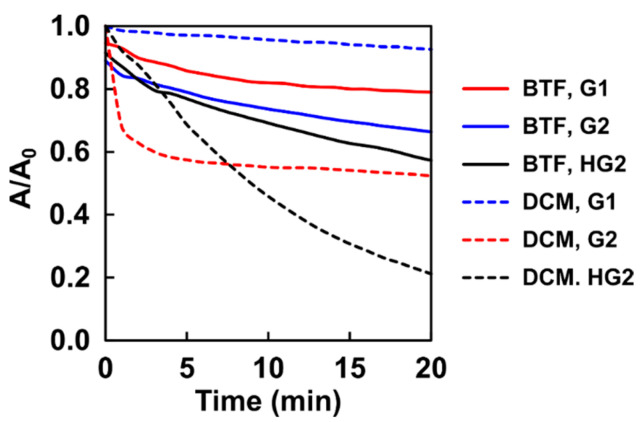
The consumption (A/A_0_) of diethyl 2,2-diallylmalonate (c_0_ = 35.6 mM) as a function of time in the ring-closing metathesis reaction in the presence of 1 mol% of ruthenium catalysts, G1, G2, and HG2 in benzotrifluoride and dichloromethane solvents at 25 °C (A: ^1^H NMR signal integral of the vinylic hydrogen signal of diethyl 2,2-diallylmalonate; and A_0_: initial ^1^H NMR signal integral of the vinylic hydrogen of diethyl 2,2-diallylmalonate; see Figure S4).

Ring-closing metathesis (RCM) transformation is also a common reaction, especially for various special ring-forming reactions in the pharmaceutical industry [65]. In order to test the suitability of BTF for this reaction, experiments were also carried out with diethyl diallylmalonate, a typical model substrate for RCM (Scheme 1, middle), in both BTF and DCM. As shown in Table 3, the rate constant in benzotrifluoride is five times higher than in dichloromethane using the G2 catalyst. The conversion is also significantly higher after 20 min; however, there is no considerable difference in the final conversion data after 24 h of reaction time. In the case of the G1 and HG2 catalysts, benzotrifluoride also proved to be a useful medium for the ring-closing metathesis, although the data in Figure 2 and Table 3 indicate that the process is somewhat slower with these catalysts in BTF than in DCM.

### 2.3. Isomerization Reaction

The olefin metathesis reactions produce mixtures of geometric isomers of alkenes, where the ratio of the E/Z isomers is highly dependent on the catalyst and solvent [65]. Stereoselective reactions are highly sought in fine chemical and pharmaceutical industrial research and development. Taking this into consideration, the effect of benzotrifluoride on the isomerization reaction was investigated using (Z)-but-2-ene-1,4-diyl diacetate as a model compound (Scheme 1, bottom). In the case of the G1, G2, and HG2 catalysts, E is the preferred isomer (Figure S5).

**Figure 3:**
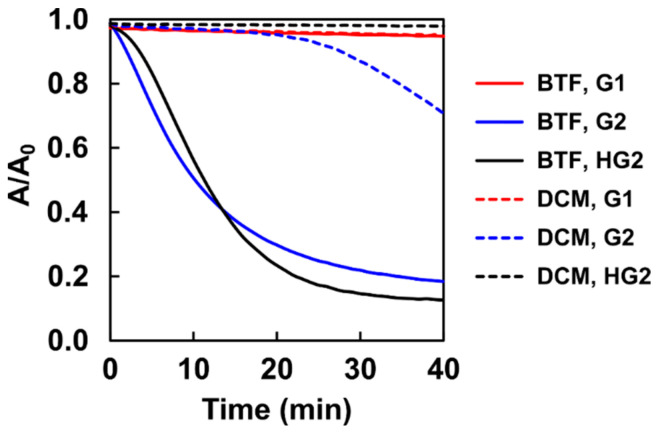
The consumption (A/A_0_) of (Z)-but-2-ene-1,4-diyl diacetate (c_0_ = 55.2 mM) as a function of time in the isomerization reaction in the presence of 0.1 mol% of ruthenium catalysts, G1, G2, and HG2 in benzotrifluoride and dichloromethane solvents at 25 °C (A: ^1^H NMR signal integral of the vinylic hydrogen signal of (Z)-but-2-ene-1,4-diyl diacetate; and A_0_: initial ^1^H NMR signal integral of the vinylic hydrogen of (Z)-but-2-ene-1,4-diyl diacetate; see Figure S5).

In the case of the reactions using G2 and HG2 as catalysts, the isomerization is much faster in BTF than in DCM in the initial period, and the rate constants of the isomerization reaction are 3.5 and 10 times higher in benzotrifluoride than in dichloromethane. However, after 72 h, the equilibrium ratios of the E/Z isomers are almost the same in both solvents. The reaction rates are very low in both solvents when the G1 catalyst is used (Figure 3 and Table 4). When the reaction is solvated with benzotrifluoride, the induction period is relatively short, while when dichloromethane is used with either the G2 or HG2 catalysts, a long induction period is observed. Nevertheless, both catalysts exhibited high conversion and were highly selective for the E isomer after 72 h of reaction time, as shown in Table 4.

## 3. Materials and Methods

### 3.1. Materials

Ruthenium catalysts, G1, G2, HG2, norbornene, diethyl 2,2-diallylmalonate (Sigma-Aldrich), and dry solvents (VWR Hungary) were used as received. Deuterated solvents were purchased from Eurisotop. Benzotrifluoride (anhydrous, ≥99%, Sigma-Aldrich) was purified as described previously [59]. Briefly, BTF was refluxed for 48 h, distilled over CaH_2_, and stored under a dry nitrogen atmosphere until used. (Z)-but-2-ene-1,4-diyl diacetate was prepared according to standard literature procedures [66].

### 3.2. Kinetic Investigations

In a nitrogen-filled glovebox, norbornene (20.0 mg, 212.8 µmol), diethyl 2,2-diallylmalonate (68.4 µL, 71.2 µmol), or (Z)-but-2-ene-1,4-diyl diacetate (82 µL, 110.3 µmol) were dissolved in 2.00 mL of dry DCM or BTF solvent. A quantity of 0.5 mL of the stock solution was transferred to a screw-capped NMR tube, whereas a deuterated water-filled capillary was inserted into the NMR tube if the solvent was not deuterated. The NMR tube was removed from the glovebox. A calculated volume of the catalyst solution (10 mg of catalyst dissolved in 0.5 mL THF previously prepared in a glovebox) was added. The in situ ^1^H NMR spectrum was obtained from the sample every 30 or 60 s.

### 3.3. Synthesis of Polynorbornene for Turnover Number Determination in BTF and DCM

The turnover number was determined using a standard protocol. A quantity of 50 mg norbornene was dissolved in 5 mL of solvent and stirred intensively in a glovebox. An amount of 2 × 10^−6^ equivalents (2 ppm) of catalyst in 5 μL THF were added. The reaction mixture was stirred for 76 h, then 122 μL of anisole was added as an internal standard. The reaction mixture was analyzed by ^1^H NMR measurements using a D_2_O-filled capillary.

### 3.4. Characterization Methods

The ^1^H NMR spectra were obtained by a Varian NMR System spectrometer operating at the ^1^H frequency of 400 MHz with a 5 mm inverse detection tunable dual-broadband {^1^H–^19^F}/{^31^P–^15^N} probe equipped with a Z-gradient. The solvent signal of CD_2_Cl_2_ (5.35 ppm on the ^1^H scale) was used as a reference for the chemical shift. In cases of non-deuterated solvents, a capillary filled with deuterated water (D_2_O) was inserted into the NMR tube as a reference (4.80 ppm on the ^1^H scale). The following parameters were used for each ^1^H measurement: number of transients = 4, recycle delay = 12.0 s, acquisition time = 3.0 s, and temperature = 25.0 °C. Each spectrum was recorded for 30 or 60 s. The spectra of the kinetic measurements were recorded in arrays of either 30 or 60 s.

The molecular weight of the polymers was determined by Gel Permeation Chromatography (GPC). The GPC system was equipped with a Waters^TM^ 515 HPLC pump, a Waters^TM^ in-line degasser, and a Waters^TM^ 717 Plus Autosampler. The columns were Waters^TM^ Styragel HR1 and HR4 in a Jetstream column thermostat, and an Agilent Infinity 390 differential refractive index detector was used. The flow rate was 0.3 mL/min at 35 °C. The evaluation was based on a calibration curve made with narrow distribution polystyrene standards. This column system is capable of separating molecular weights of up to 600,000 g/mol, but we concluded that the molecular weight of our products was above this region, and we therefore could not achieve a full separation during the measurements. For this reason, we feel that the data in Table 1 underestimates the real results. It has also been previously shown that the polystyrene calibration yields molecular weights that are two times higher than the actual ones [67], but as we could not fully separate the molecules, we set this aside for further calculation.

## 4. Conclusions

Systematic comparative experiments were carried out to determine whether the environmentally friendly benzotrifluoride (BTF) can be used as a solvent in olefin metathesis reactions such as ring-opening metathesis polymerization (ROMP), ring-closing metathesis polymerization (RCM), and isomerization, rather than the hazardous, commonly used dichloromethane (DCM). Benzotrifluoride gave excellent results for the ring-opening metathesis polymerization, resulting in polynorbornene with high yields and high molecular weights (M_n_ > 100,000 g/mol). The kinetic parameters of BTF are similar to those of the more frequently used DCM. The ROMP of norbornene in BTF had excellent turnover numbers, i.e., in the range of 380,000–490,000 using only 2 ppm of catalysts. This clearly indicates that BTF, as an environmentally advantageous solvent, is an outstanding and suitable replacement for the environmentally dangerous DCM in ring-opening metathesis polymerization reactions.

High conversions were achieved in the ring-closing metathesis (RCM) reaction of diethyl 2,2-diallylmalonate in benzotrifluoride, which proved that this solvent is also a suitable medium for RCM reactions. In addition, in the case of the isomerization reaction of (Z)-but-2-ene-1,4-diyl diacetate using the G2 and HG2 catalysts, much faster initial isomerization occurs in BTF than in DCM, and significantly shorter induction periods are observed in BTF than in DCM.

Based on our results, it can be concluded that benzotrifluoride can find numerous applications as an alternative, affordable, and sustainable solvent for olefin metathesis processes with high efficiency, not only in academic research but in industrial applications as well, where the use of a non-toxic and less volatile solvent is highly recommended.

## Figures and Tables

**Table 1:** The catalyst contents, rate constant (k_1_), and half-life (t_1/2_), determined by fitting the data of monomer consumption in the first 3 min, and the average molecular weight (M_n_) of the polymers obtained in the ring-opening metathesis polymerization of norbornene in benzotrifluoride (BTF) and dichloromethane (DCM) solvents at 25 °C.

Solvent	Catalyst (mol%)	k_1_ (min^−1^)	t_1/2_ (min)	M_n_·10^−3^ (g/mol) *
BTF	G1 (0.050)	0.82	0.85	138.7
DCM	G1 (0.050)	0.79	0.88	157.0
BTF	G2 (0.010)	0.72	0.96	106.0
DCM	G2 (0.010)	0.91	0.76	245.1
BTF	HG2 (0.003)	0.66	1.05	175.8
DCM	HG2 (0.003)	1.26	0.55	363.5

* Determined using GPC (samples were prepared with a 0.05 mol% catalyst).

**Table 2:** The solvent, the catalyst type, the conversion, and the turnover number for the ring-opening metathesis polymerization of norbornene (c_0_ = 106 mM) conducted in benzotrifluoride and dichloromethane under a nitrogen atmosphere for 78 h.

Solvent	Catalyst	Conversion *	Turnover Number *
BTF	G1	0.77	385,100
DCM	G1	0.95	477,100
BTF	G2	>0.99	499,900
DCM	G2	>0.99	499,500
BTF	HG2	0.96	481,200
DCM	HG2	>0.99	499,800

* Determined by ^1^H NMR measurements using anisole as an internal standard.

**Table 3:** The solvent, the catalyst, rate constant, and the conversions after 20 min and 24 h in the ring-closing metathesis reactions of diethyl 2,2-diallylmalonate (c_0_ = 35.6 mM) in benzotrifluoride (BTF) and dichloromethane (DCM) using 1 mol% G1, G2, or HG2 as catalysts.

Solvent	Catalyst	k_1_ (min^−1^)	Conversion at 20 min (%)	Conversion at 24 h (%)
BTF	G1	0.019	21	55
DCM	G1	0.202	47	>99
BTF	G2	0.024	34	96
DCM	G2	0.005	7	>99
BTF	HG2	0.033	43	80
DCM	HG2	0.075	78	>99

**Table 4:** The solvent, the catalyst, rate constant, and the conversions after 20 min and 24 h of the isomerization of (Z)-but-2-ene-1,4-diyl diacetate (c_0_ = 55.2 mM) in benzotrifluoride (BTF) and dichloromethane (DCM) using 0.1 mol% G1, G2, and HG2 as catalysts.

Solvent	Catalyst	k_1_ (min^−1^)	Conversion at 20 min (%)	Conversion at 72 h (%)
BTF	G1	<0.001	4	19
DCM	G1	<0.001	4	20
BTF	G2	0.070	70	89
DCM	G2	0.021	5	93
BTF	HG2	0.092	77	88
DCM	HG2	0.009	2	93

## Data Availability

Additional data on polymer analyses can be obtained on request from the corresponding authors.

## Supplementary Materials

The following supporting information can be downloaded at: https://www.mdpi.com/article/10.3390/ijms24010671/s1.
