# Supplementary material for: Ring-Opening Metathesis Polymerization and Related Olefin Metathesis Reactions in Benzotrifluoride as an Environmentally Advantageous Medium
Source: Int J Mol Sci. 2022 Dec 30;24(1):671. doi: 10.3390/ijms24010671 (PMC9820898; doi:10.3390/ijms24010671)
Supplement: Supplementary file 1 [file ijms-24-00671-s001.zip › ijms-2118803-supplementary.pdf]

Supporting information

Ring-Opening Metathesis Polymerization and Related Olefin Metathesis Reactions in Benzotrifluoride as an Environmentally Advantageous Medium

Ervin Kovács[1*], Bence Balterer[2,3], Nguyen Anh Duc[1], Györgyi Szarka[1], Michael C. Owen[4], Attila Domján[2,*] and Béla Iván[1]

[1]Polymer Chemistry and Physics Research Group, Institute of Materials and Environmental Chemistry, Research Centre for Natural Sciences, Magyar tudósok Krt. 2, H-1117 Budapest, Hungary

[2]NMR Research Laboratory, Centre for Structural Science, Research Centre for Natural Sciences, Magyar tudósok krt. 2, H-1117 Budapest, Hungary

[3]Hevesy György PhD School of Chemistry, Eötvös Loránd University, Pázmány sétány 1, H-1117 Budapest, Hungary

[4]Institute of Chemistry, University of Miskolc, H-3515 Miskolc-Egyetemváros, Hungary

*Correspondence: domjan.attila@ttk.hu

† In memory of Professor Robert H. Grubbs.

CONTENT

Figure S1. GPC traces of the polymers prepared by ROMP of norbornene in benzotrifluoride (left) and dichloromethane (right) using G1, G2 or HG2 catalyst at 25 °C.

Figure S2. Stacked ^{1}H NMR spectra of the ROMP of norbornene using 0.001 mol% of G2 catalyst in benzotrifluoride at 25 °C in every minute from 1 minute to 10 minutes.

Figure S3. Stacked ^{1}H NMR spectra of the ROMP of norbornene using 0.001 mol% of G2 catalyst in CD_2Cl_2 at 25 °C in every two minutes from 1 minute to 39 minutes.

Figure S4. Stacked ^{1}H NMR spectra of the RCM reaction of diethyl diallylmalonate using 1 mol% G2 catalyst in benzotrifluoride at 25 °C in every six minutes.

Figure S5. Stacked ^{1}H NMR spectra of isomerization reaction of (Z)-but-2-ene-1,4-diyl diacetate using 0.001 equivalent of G2 catalyst in benzotrifluoride in every six minutes.

Table S1. The solvent, the atmosphere, the catalyst type, the conversion and the turnover number for ROMP of norbornene conducted in benzotrifluoride at various conditions using 2 ppm catalyst at room temperature for 78 hours.

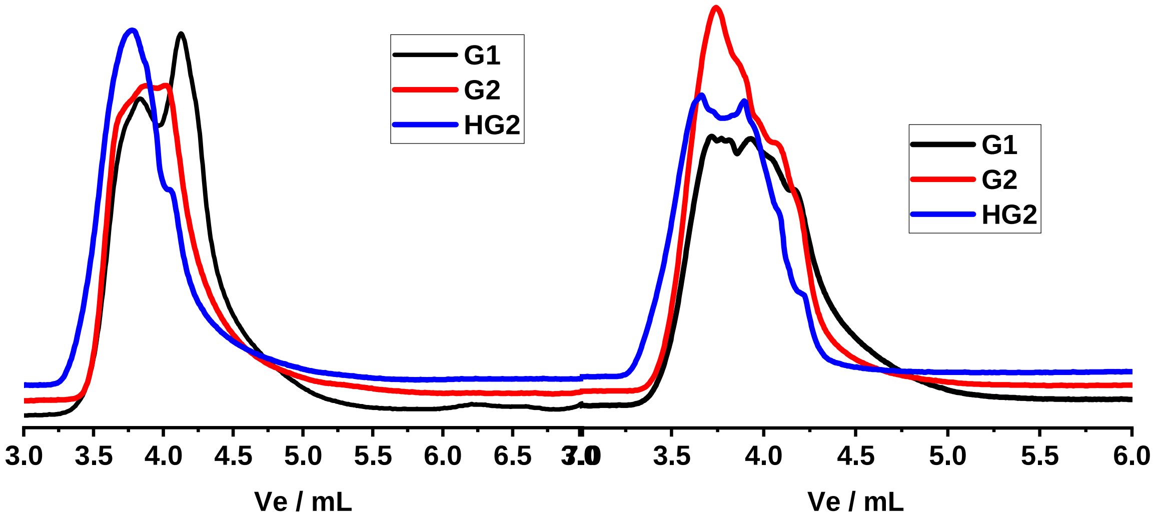

Figure S1. GPC traces of the polymers prepared by ROMP of norbornene in benzotrifluoride (left) and dichloromethane (right) using G1, G2 or HG2 catalyst at 25 °C. GPC measurements were carried out in THF at 35 °C.

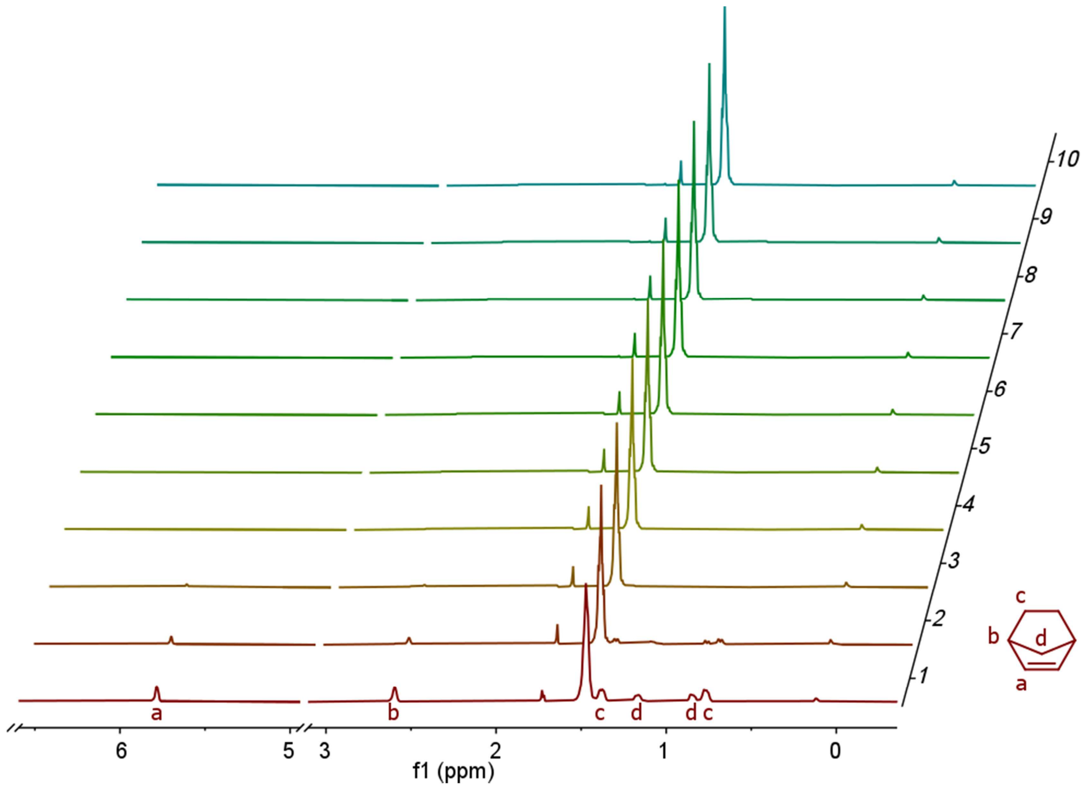

Figure S2. Stacked ^{1}H NMR spectra of the ring-opening metathesis polymerization reaction of norbornene (c_0 = 106 mM) using 0.001 mol% of G2 catalyst in benzotrifluoride at 25 °C in every minute from 1 minute (bottom) to 10 minutes (top).

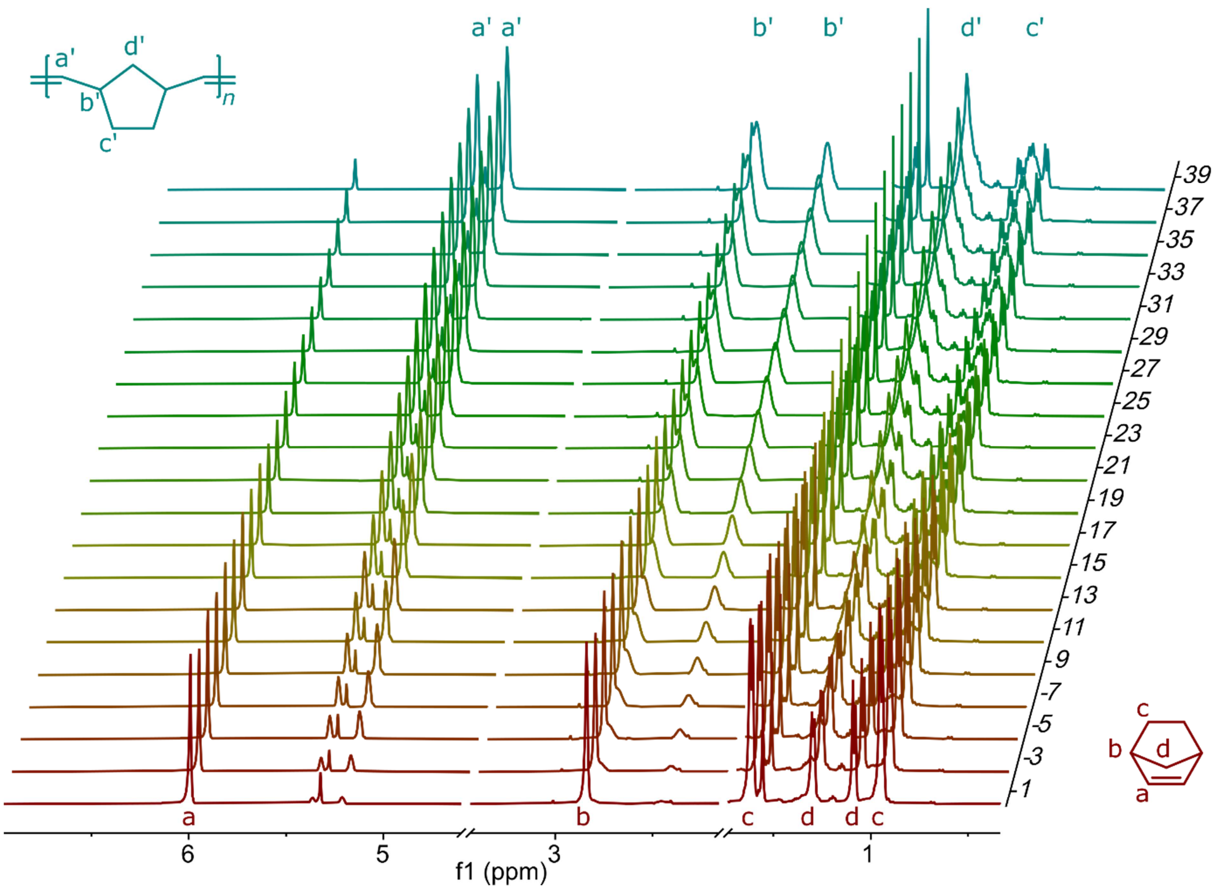

Figure S3. Stacked ^{1}H NMR spectra of the ROMP of norbornene ($c_0 = 106$ mM) using 0.001 mol% of G2 catalyst in CD_2Cl_2 at 25 °C in every two minutes from 1 minute (bottom) to 39 minutes (top).

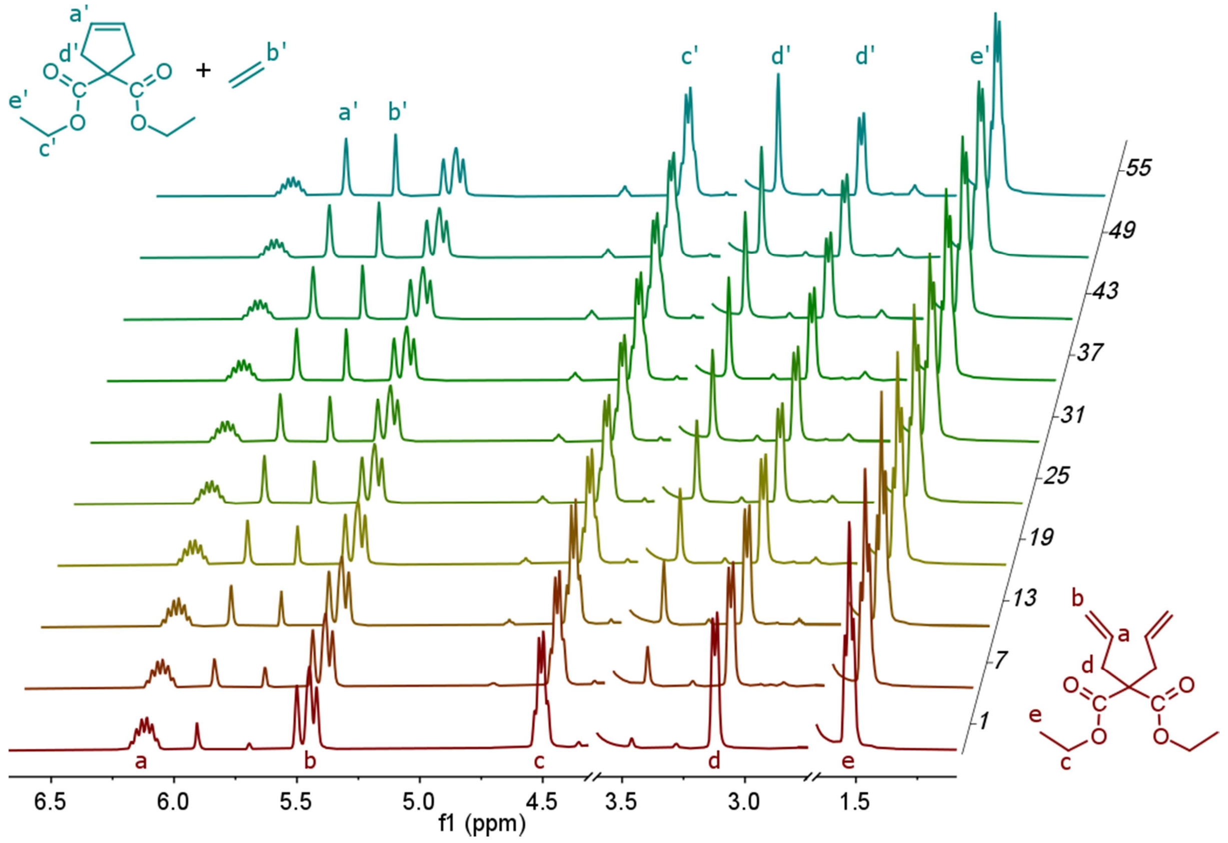

Figure S4. Stacked ^{1}H NMR spectra of the RCM reaction of diethyl diallylmalonate (c_0 = 35.6 mM) using 1 mol% G2 catalyst in benzotrifluoride at 25 °C in every six minutes from 1 minute (bottom) to 55 minutes.

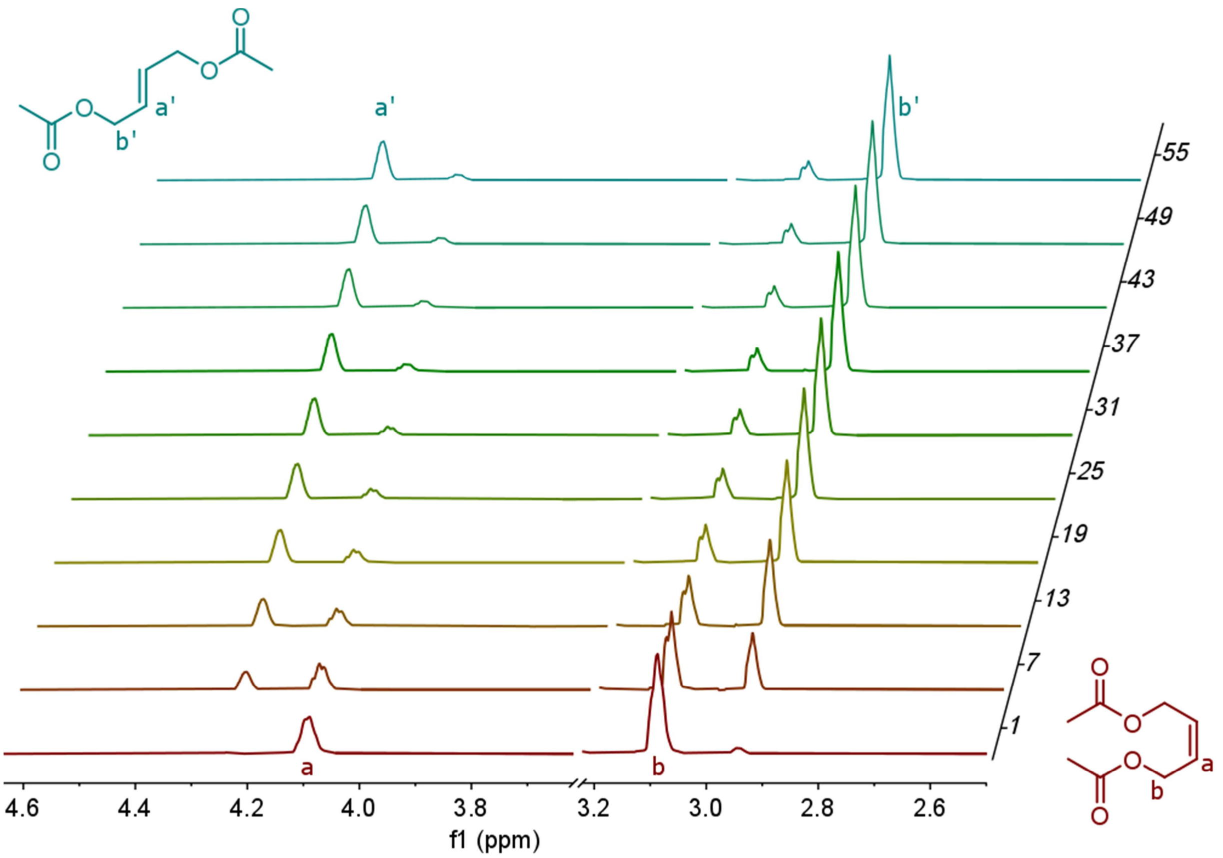

Figure S5. Stacked ^{1}H NMR spectra of isomerization reaction of (Z)-but-2-ene-1,4-diyl diacetate (c_0 = 55.2 mM) using 0.001 equivalent of G2 catalyst in benzotrifluoride in every six minutes from 1 minute (bottom) to 55 minutes (top).

Table S1. The solvent, the atmosphere, the catalyst type, the conversion and the turnover number for ROMP of norbornene (c_0 = 106 mM) conducted in benzotrifluoride at various conditions using 2 ppm catalyst at room temperature for 78 hours.

Solvent	Atmosphere	Catalyst	Conversion*	Turnover number*
Distilled BTF	Nitrogen	G1	0.77	385,100
Crude BTF	Nitrogen	G1	0.39	197,300
Crude BTF	Air	G1	0.15	75,300
Distilled BTF	Nitrogen	G2	>0.99	499,900
Crude BTF	Nitrogen	G2	0.95	474,300
Crude BTF	Air	G2	0.19	94,300
Distilled BTF	Nitrogen	HG2	0.96	481,200
Crude BTF	Nitrogen	HG2	0.70	352,300
Crude BTF	Air	HG2	0.33	162,600

*Determined by ^{1}H NMR measurements using anisole as internal standard.
